# Comparative Diagnostic Accuracy of Contrast-Enhanced Ultrasound and Shear Wave Elastography in Differentiating Benign and Malignant Lesions: A Network Meta-Analysis

**DOI:** 10.3389/fonc.2019.00102

**Published:** 2019-03-05

**Authors:** Rongzhong Huang, Lihong Jiang, Yu Xu, Yuping Gong, Haitao Ran, Zhigang Wang, Yang Sun

**Affiliations:** ^1^The First People's Hospital of Yunnan Province, Kunming, China; ^2^Chuangxu Institute of Life Science, Chongqing, China; ^3^Second Affiliated Hospital of Chongqing Medical University, Chongqing, China

**Keywords:** contract enhanced ultrasonography, malignant lesions benign lesions, network meta analysis, shear wave elastography, lesions

## Abstract

**Background:** We performed a network meta-analysis to compare the diagnostic accuracy of contrast-enhanced ultrasound (CEUS) and shear wave elastography (SWE) in differentiating benign and malignant lesions in different body sites.

**Methods:** A computerized literature search of Medline, Embase, SCOPUS, and Web of Science was performed using relevant keywords. Following data extraction, we calculated sensitivity, specificity, positive likelihood ratio (LR), negative LR, and diagnostic odds ratio (DOR) for CEUS, and SWE compared to histopathology as a reference standard. Statistical analyses were conducted by MetaDiSc (version 1.4) and R software (version 3.4.3).

**Results:** One hundred and fourteen studies (15,926 patients) were pooled in the final analyses. Network meta-analysis showed that CEUS had significantly higher DOR than SWE (DOR = 27.14, 95%CI [2.30, 51.97]) in breast cancer detection. However, there were no significant differences between CEUS and SWE in hepatic (DOR = −6.67, 95%CI [−15.08, 1.74]) and thyroid cancer detection (DOR = 3.79, 95%CI [−3.10, 10.68]). Interestingly, ranking analysis showed that CEUS achieved higher DOR in detecting breast and thyroid cancer, while SWE achieved higher DOR in detecting hepatic cancer. The overall DOR for CEUS in detecting renal cancer was 53.44, 95%CI [29.89, 95.56] with an AUROC of 0.95, while the overall DOR for SWE in detecting prostate cancer was 25.35, 95%CI [7.15, 89.89] with an AUROC of 0.89.

**Conclusion:** Both diagnostic tests showed relatively high sensitivity and specificity in detecting malignant tumors in different organs. Network meta-analysis showed that CEUS had higher diagnostic accuracy than SWE in detecting breast and thyroid cancer, while SWE had higher accuracy in detecting hepatic cancer. However, the results were not statistically significant in hepatic and thyroid malignancies. Further head-to-head comparisons are needed to confirm the optimal imaging technique to differentiate each cancer type.

## Introduction

Ultrasound (US) has been used for decades in differentiating benign and malignant lesions because of its low cost, ease of access, and non-invasiveness. For example, it belongs to the triad (physical examination, mammography and US), commonly used to assess the risk of breast cancer (1). Moreover, it can detect thyroid nodules as small as 2 mm in size and predicts malignancy based on features like irregular border, hypo-echogenicity, and calcification (2, 3). However, none of these features can individually predict malignancy and conventional US alone has shown moderate accuracy in detecting malignant lesions (4). Therefore, improvements to US technique have been sought.

The introduction of contrast agents (contrast-enhanced US/CEUS) allows for visibility of blood flow within the lesion, which improves its characterization (5). The current in-use contrast media are second-generation agents as SonoVue. These agents remain within the intravascular space, which increases their safety and allows for continuous imaging over the enhancement period (6). Several studies have reported high sensitivity and specificity for CEUS in differentiating malignant lesions with the breast, thyroid, liver and kidneys (5, 7–9). A recent meta-analysis showed no significant difference between CEUS and contrast-enhanced computed tomography (CECT) and magnetic resonance imaging (CEMRI) in terms of the diagnostic accuracy in characterizing focal liver lesions (FLLs) (8).

Shear wave elastography (SWE) relies on the degree of lesion stiffness when subjected to external pressure. Malignant nodules have harder consistency (less elasticity) than benign ones due to the uncontrolled proliferation of cancer cells (10). Therefore, SWE has been investigated for differentiating benign and malignant nodules. Compared to conventional US, SWE is more quantitative and is less operator-dependent, allowing more effective detection of malignant tumors (11). Recent diagnostic test accuracy (DTA) studies and meta-analyses showed high sensitivity and specificity for SWE in detecting malignant lesions within the breast and hepatic tissues (11–13).

According to our knowledge, data are lacking on the direct comparison between CEUS and SWE; therefore, we performed a meta-analysis to evaluate the diagnostic accuracy of CEUS and SWE in differentiating malignant tumors in the breast, liver, thyroid, kidneys, and prostate tissues in comparison to histopathology as a reference test. Moreover, we used network meta-analysis (NMA) to compare the diagnostic accuracy of both tests in malignant tumor differentiation.

## Materials and Methods

This meta-analysis has been conducted and reported in accordance with the Preferred Reporting Items for a Systematic Review and Meta-analysis of Diagnostic Test Accuracy Studies (The PRISMA-DTA Statement) (14); Supplementary File I.

### Literature Search

We searched Medline (via PubMed), Embase, SCOPUS and Web of Science for diagnostic accuracy studies that evaluated the use of CEUS and SWE in the differentiation of malignant tumors in different body organs. The following search terms were used with different combinations in different databases: Contrast-enhanced Ultrasound OR CEUS OR Ultrasound OR SonoVue OR Shear Wave Elastography OR SWE OR Sonoelastography OR Elastosonography AND Malignant OR Cancer OR Tumor OR Benign OR Adenoma OR Adenocarcinoma OR Carcinoma OR Nodule. No search filters of any sort were used during the search. All retrieved search results from database search (including bibliographic data and abstracts) were imported into EndNote (X7) for duplicate removal and then were transferred to a Microsoft Excel Sheet for screening.

### Study Screening

For a study to be eligible for inclusion, it must have matched all the following criteria: (1) Population: Patients, suspected or diagnosed with malignancy in any body organ, (2) Intervention: CEUS or SWE [no specifications by US system or probe type], (3) Comparator: Histopathology, (4) Outcomes: Sensitivity, specificity, positive predictive value [PPV], and negative predictive value [NPV], and (5) Study type: Diagnostic accuracy study. Two independent authors reviewed the title and abstract of retrieved records against our eligibility criteria and classified them into: eligible, non-eligible, or requires further screening (seems to fit the inclusion criteria, but further confirmation is required). The full-text articles of the latter type were retrieved and underwent a second wave of screening. Any discrepancy between the two reviewers' decisions was solved by a senior reviewer (with a 15-year experience in secondary analysis and evidence synthesis methods) after reviewing the debated studies in reference to the pre-specified PICO criteria.

### Data Extraction and Quality Assessment

An extraction sheet (in Microsoft Excel) was formatted and pilot-tested before final extraction. The sheet was customized to extract the baseline data of the imaging device, enrolled patients, as well as the raw diagnostic data of each included study. For pilot testing, two reviewers extracted these data from 5 included studies and the datasets were matched and compared with the original studies by a third reviewer. Each set of data was extracted by two reviewers and discordant decisions were resolved by discussion. These discussions included re-examining the studies, inspecting their available additional data sources and re-evaluating the former decisions. When the discrepancies remained, a senior reviewer examined the studies and settled the differences. The extracted data included (I) baseline characteristics of enrolled participants, (II) study design, (III) diagnostic test parameters: Parameters, cutoff value and US system for SWE and contrast agent, US technique, probe and mechanical index for CEUS, and (IV) Outcome data: true positive (TP), true negative (TN), false positive (FP), and false negative (FN) values. When these values were not directly given, they were calculated from the processed data as sensitivity, specificity, PPV, and NPV, using the statistical calculator on RevMan software (Version 5.3 for Windows). We used the Quality Assessment of Diagnostic Accuracy Studies (QUADAS) score to assess the quality of included studies. It consists of 14 (yes/no/unclear) questions to assess different forms of bias within DTA studies (15).

### Data Analysis

Pairwise meta-analyses were done under the random-effects model when two or more studies investigated the same predefined research question with the same laboratory test. We extracted the sensitivity, specificity, positive likelihood ratio (LR), negative LR, and diagnostic odds ratio (DOR) values for CEUS and SWE compared to histopathology as a reference standard. The DOR is calculated as (TP X TN)/ (FP X FN) and defined as the odds of having a positive test result in a patient with disease compared with the odds of a positive test result in a patient without disease. Moreover, summary receiver operating characteristic (SROC) curves were constructed to examine diagnostic accuracy. All statistics were reported as absolute values with their 95% confidence interval (95% CI). A *p*-value < 0.05 was considered statistically significant. The Chi-square and I-square statistics were calculated in order to assess heterogeneity. Significant heterogeneity was considered to be present if the chi-square *p*-value was < 0.1 (as per the Cochrane Handbook for Systematic Reviews of Intervention). Data were presented into five subgroups according to cancer site: breast, liver, thyroid, kidneys, and prostate. Network meta-analyses were conducted to compare the diagnostic accuracy of CEUS vs. SWE in malignancy detection. Heterogeneity and inconsistency were checked by the I^2^ and the corresponding *p*-value. All statistical analyses were conducted on MetaDiSc (version 1.4) and R software (version 3.4.3).

## Results

### Literature Search and Study Characteristics

**Figure 1:**
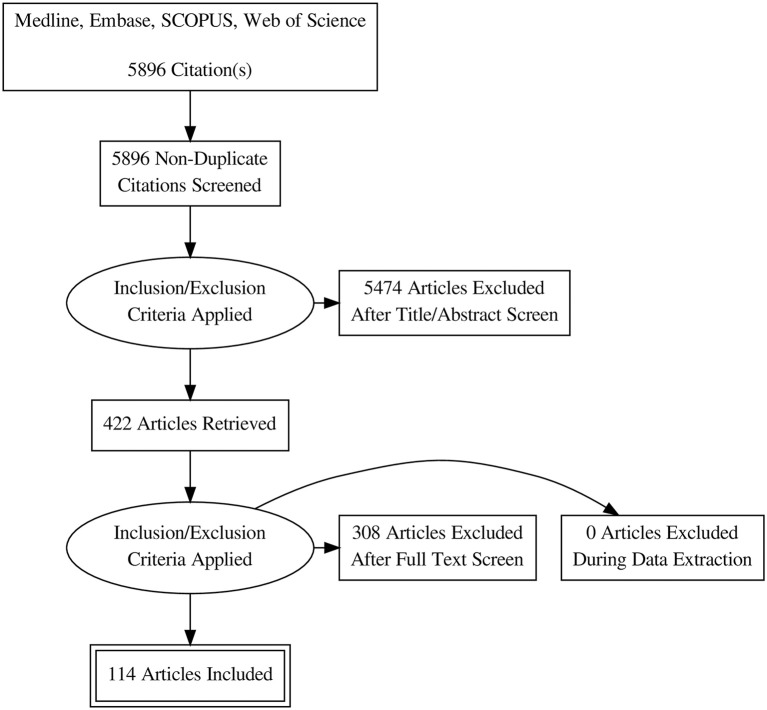
PRISMA flow diagram of literature search and study selection.

Database search retrieved 5896 unique citations. Following title and abstract screening, 422 full-text articles were retrieved for further scrutiny. Finally, 114 diagnostic accuracy studies (65 on SWE and 50 on CEUS; one study by 4 assessed both modalities), reporting data from 15926 patients (5680 for CEUS and 10392 for SWE) were included in our network meta-analysis (Figure 1, Bibliographic details in Supplementary File II). According to the QUADAS score, 25 (21.5%), 30 (25.8%), 22 (18.9%), 23 (19.8%), and 16 (13.8%) studies scored 10, 11, 12, 13, and 14, respectively. The baseline data of enrolled participants, as well as the characteristics of the used US systems for SWE and CEUS tests are illustrated in Tables 1, 2, respectively.

**Table 1:** Baseline characteristics of enrolled patients and criteria of the used SWE system.

**References**	**Country**	**Study design**	**Patients/Lesions (N)**	**Age (Years)**	**Male: Female**	**Organ**	**Condition**	**Reference test/Gold standard**	**SWE parameters**	**Cutoff value (Kpa)**	**US system**
Li et al. (16)	China	Prospective cohort	276 (296 lesions)	45.4 ± 14.7	100% F	Breast	Benign vs. malignant breast masses	Histopathology	SWS	4.39 m/sec	S3000 US scanner (Siemens)
Yang et al. (17, 18)	China	Retrospective cohort	218 (225 lesions)	45.3 ± 14.6	100% F	Breast	Benign vs. malignant breast masses	Histopathology	Emean	36.1 Kpa	Aplio500 US machine (Toshiba)
Elmoneam et al. (13)	Egypt	Prospective cohort	63 (63 lesions)	34.7 ± 5.9	100% F	Breast	Benign vs. malignant breast masses	Histopathology	Emax	106.55 Kpa	N/A
Kim et al. (19)	Korea	Retrospective cohort	171 (177 lesions)	45.17 ± 9.37	100% F	Breast	Small breast lesions < 2 cm	Histopathology	Emax	87.5 Kpa	Aixplorer system (Supersonic Imagine
Youk et al. (20)	Korea	Prospective cohort	123 (130 lesions)	46.7 ± 11.2	100% F	Breast	Breast cancer	Histopathology	Emean	82.2 Kpa	Aixplorer ultrasound system
Tang et al. (21)	China	Prospective cohort	98 (133 lesion)	N/A	100% F	Breast	Benign vs. malignant breast lesion	Histopathology	Mean SWV	3.68 m/s	Siemens S3000 US scanner
Choi et al. (22)	Korea	Retrospective cohort	54 (56 lesions)	40.76 + 68.07	100% F	Breast	Benign vs. malignant breast lesion	Histopathology	Emean	44.3 Kpa	Aixplorer US system (SuperSonic Imagine
Liu et al. (12)	China	Prospective cohort	130 (139 lesions)	44.74 ± 14.77	100% F	Breast	Benign vs. malignant breast lesion	Histopathology	Max SWV	5.37 m/s	Siemens Acuson S3000 ultra-sound machine
Golatta et al. (23)	Germany	Prospective cohort	103 (104 lesions)	51 ± 18.56	100% F	Breast	Benign vs. malignant breast lesion	Histopathology	Mean SWV	5.18 m/s	Siemens Medical Solutions
Youk et al. (24)	Korea	Retrospective cohort	324 (389 lesions)	46.0 ± 11.4	100% F	Breast	Benign vs. malignant breast lesion	Histopathology	Eratio	5.14	Aixplorer US system (SuperSonic Imagine
Ko et al. (25)	Korea	Retrospective cohort	33 (34 lesions)	46.4 ± 7.5	100% F	Breast	Breast Non-mass lesions	Histopathology	Emean	41.6 Kpa	Aixplorer US system (SuperSonic Imagine
Lee et al. (26)	Korea	Prospective cohort	134 (144 lesions)	49.1 ± 12.8	100% F	Breast	Benign vs. malignant breast lesion	Histopathology	Emax	147.2 Kpa	Aixplorer US system (SuperSonic Imagine
Ng et al. (27)	Malaysia	Prospective cohort	152 (159 lesions)	52 + 20.5	100% F	Breast	Benign vs. malignant breast lesion	Histopathology	Emax	56.0 Kpa	Aixplorer ultrasound system (SuperSonic Imagine
Tian et al. (28)	China	Retrospective cohort	210 (210 lesions)	43.12 ± 13.34	100% F	Breast	Benign vs. malignant breast lesion	Histopathology	Emax	80.8 Kpa	Aixplorer ultrasound system (SuperSonic Imagine
Olgun et al. (29)	Turkey	Prospective cohort	109 (115 lesions)	51 + 17.5	0.02:1	Breast	Benign vs. malignant breast lesion	Histopathology	Eratio	4.7	Aixplorer ultrasound system (SuperSonic Imagine
Chang et al. (30)	Korea	Prospective cohort	115 (133 lesions)	51.4 + 11.75	100% F	Breast	Benign vs. malignant breast lesion	Histopathology	Emean	60.7 Kpa	IU-22 (Phillips) OR HDI 5000 sonography unit
Yao et al. (31)	China	Prospective cohort	206 (206 lesions)	44.6 + 13.3	100% F	Breast	Benign vs. malignant breast lesion	Histopathology	Mean SWV	4.22 m/s	Acuson S2000 ultrasound system (Siemens
Lee et al. (26)	Korea	Retrospective cohort	139 (156 lesions)	43.54 ± 9.94	100% F	Breast	Solid breast masses	Histopathology	Emax	82.3 Kpa	Aixplorer ultrasound system (SuperSonic Imagine
Seo et al. (32)	Korea	Prospective cohort	37 (45 lesions)	47.4 +14.75	100% F	Breast	Benign vs. malignant breast lesion	Histopathology	Emean	67.8 Kpa	Aplio 500; Toshiba
Au et al. (33)	Canada	Prospective cohort	112 (123 lesions)	49.2+10.7	100% F	Breast	Solid breast masses	Histopathology	Eratio	3.56	Aixplorer Multiwave V3, Supersonic Imagine
Chang et al. (34)	Korea	Prospective cohort	129 (150 lesions)	47.8+8.83	100% F	Breast	Benign vs. malignant solid breast lesions	Histopathology	Emean	80 Kpa	Aixplorer, SuperSonic Imagine
Choi et al. (35)	Korea	Retrospective cohort	113 (116 lesions)	48.4+10	100% F	Breast	Breast non-mass lesions	Histopathology	Emean	85.1 Kpa	Aixplorer, SuperSonic Imagine
Chung et al. (36)	Korea	Retrospective cohort	71 (79 lesions)	48+10.67	100% F	Breast	Breast papillary lesions	Histopathology	Emax	62.1 Kpa	Aixplorer, SuperSonic Imagine
Choi et al. (22)	Korea	Retrospective cohort	199 (205 lesions)	51.7 ± 13.3	100% F	Breast	Benign vs. malignant solid breast lesions	Histopathology	Emean	85.8 Kpa	Aixplorer, SuperSonic Imagine
Dobruch-Sobczak et al. (37)	Poland	Retrospective cohort	76 (84 lesions)	59.9+13	100% F	Breast	Focal breast lesions	Histopathology	Eav.adj.	68.5 Kpa	Aixplorer, SuperSonic Imagine
Guo et al. (38)	China	Prospective cohort	379 (404 lesions)	N/A	100% F	Breast	Focal breast lesions	Histopathology	SWS	3.015 m/s	Siemens ACUSON S2000
Hong et al. (39)	Korea	Prospective cohort	218 (264 lesions)	46.4+10.5	100% F	Breast	Solid breast masses	Histopathology	Emax	44.1 Kpa	N/A
Kim et al. (40)	China	Retrospective cohort	67 (67 lesions)	41.5+2.29	100% F	Breast	Fibroadenoma vs. phylloids tumor	Histopathology	Emean	43.9 Kpa	Aixplorer, SuperSonic Imagine
Klotz et al. (41)	France	Retrospective cohort	142 (167 lesions)	57.7 +11	100% F	Breast	Benign vs. malignant solid breast lesions	Histopathology	Emax	106 Kpa	Aixplorer, SuperSonic Imagine
Lee et al. (42)	Korea	Retrospective cohort	139 (140 lesions)	45.5 + 10.33	100% F	Breast	Complex cystic and solid breast lesions	Histopathology	Emax	108.5 Kpa	Aixplorer, SuperSonic Imagine
Li et al. (16)	China	Retrospective cohort	116 (116 lesions)	48.56+ 14.4	100% F	Breast	Breast lesions BIRADS IV	Histopathology	SWS	3.49 m/s	Siemens S3000 US machine
Shi et al. (43)	China	Prospective cohort	251 (279 lesions)	45.3 6 11.8	100% F	Breast	Benign vs. malignant solid breast lesions	Histopathology	SD	8.05 Kpa	Aixplorer, SuperSonic Imagine
Sim et al. (44)	UK	Retrospective cohort	52 (52 lesions)	67	100% F	Breast	IDC	Histopathology	Emean	50 Kpa	Aixplorer, SuperSonic Imagine
Sim et al. (44)	UK	Retrospective cohort	52 (52 lesions)	67	100% F	Breast	ILC	Histopathology	Emean	50 Kpa	Aixplorer, SuperSonic Imagine
Wu et al. (45)	China	Retrospective cohort	192 (209 lesions)	N/A	100% F	Breast	Benign vs. malignant solid breast lesions	Histopathology	N/A	N/A	Siemens ACUSON S2000
Youk et al. (20)	Korea	Retrospective	78 (79 lesions)	45.5 + 11.6	100% F	Breast	Benign vs. malignant solid breast lesions	Histopathology	Eratio	3.7	Aixplorer, SuperSonic Imagine
Zhang et al. (46)	China	Prospective cohort	97 (98 lesions)	44.74 ± 14.77	100% F	Breast	Small breast lesions < 10 cm	Histopathology	SWV	3.27 m/s	Siemens ACUSON S2000
Cong et al. (47)	China	Prospective cohort	315 (326 lesions)	44.51 + 11.81	100% F	Breast	Breast masses	Histopathology	SD	13.75	Aixplorer, SuperSonic Imagine
Park et al. (48, 49)	Korea	Retrospective cohort	133 (156 lesions)	47.8 ± 12.7	100% F	Breast	Palpable breast masses	Histopathology or periodic imaging surveillance	Emax	45.1 Kpa	Aixplorer, SuperSonic Imagine
Wang et al. (50)	China	Retrospective cohort	63 (67 lesions)	40.1 + 21.2	100% F	Breast	Non-mass breast lesions	Histopathology	Emax	81.07 Kpa	iU22 Philips
Kasai et al. (51)	Japan	Prospective cohort	273 patients with chronic liver disease	59.64 ± 14.40 70.98 ± 9.33	1:01	Liver	HCC	Histopathology	Young's Modulus	N/A	Aixplorer US system (SuperSonic Imagine S.A.)
Gerber et al. (52)	Germany	Prospective cohort	106 (106 lesions)	55.5+16.74	3.8:1	Liver	Characterization of solid HFLs	Histopathology and CE imaging for benign lesions	Emedian	37.6 Kpa	Aixplorer ultrasound system (SuperSonic Imagine)
Özmen et al. (53)	Turkey	Prospective cohort	20 (20 lesions)	4.74+4	2.3:1	Liver	Heamangioma vs. malignant liver lesions	Histopathology	Emean	23.62 Kpa	Aixplorer ultrasound system (SuperSonic Imagine)
Tian et al. (54)	China	Prospective cohort	221 (229 lesions)	48.9 + 13.2	2.4:1	Liver	Benign vs. malignant HFLs	Histopathology	Emax	39.6 Kpa	Aixplorer, SuperSonic Imagine
Ahmad et al. (55)	UK	Prospective cohort	50 (11 with PSA> 20)	69	100% M	Prostate	Prostate cancer	Histopathology	Shear wave velocity and Young's modulus	N/A	SuperSonic Imagine
Boehm et al. (56)	Germany	Prospective cohort	60 patients with suspected prostate cancer	N/A	100% M	Prostate	Prostate cancer	histopathology	Young's Modulus	50 Kpa	TRUS Aixplorer
Porsch et al. (57)	Germany	Prospective cohort	69 (794 samples)	65+8	100% M	Prostate	Prostate cancer	Histopathology	Young's Modulus	48 Kpa	SuperSonic Imagine Ultrasound System AIXPLORER
Woo et al. (58)	Korea	Prospective cohort	87 (87 lesions)	66 ± 9.0	100% M	Prostate	Prostate cancer	Histopathology	Young's Modulus	43.9 Kpa	SuperSonic Imagine
Correas et al. (59)	France	Prospective cohort	184 (1040 samples)	65.1 6 7.6	100% M	Prostate	Prostate cancer	Histopathology	Young's Modulus	35 Kpa	SuperSonic Imagine
Glybochko et al. (60)	Russia	Prospective cohort	302 (134 with suspected PC, 120 with confirmed PC and 48 healthy men)	N/A	100% M	Prostate	Prostate cancer	Histopathology	Young's Modulus	50 Kpa	Super Sonic Imagine
Zhang et al. (61, 62)	China	Prospective cohort	59 (71 lesions)	50.5 ± 9.1	0.4:1	Thyroid	Benign vs. malignant thyroid nodules < 10 mm	Histopathology	Shear wave velocity	2.910 m/s	Acuson S2000 Seimens VTTQ
Azizi et al. (63)	USA	Prospective cohort	676 (707 lesions)	51.2+15	0.2:1	Thyroid	Thyroid cancer	Histopathology	Shear wave velocity	3.54 m/s	Virtual Touch IQ Software on the Siemens ACU-SON S3000 US
Liu et al. (12)	China	Prospective cohort	271 (331 lesions)	45.9 ± 13.4	0.3:2	Thyroid	Malignant thyroid nodule	Histopathology	SWE mean	39.3 Kpa	SuperSonic Imagine
Wang et al. (64)	China	Prospective cohort	322 (322 nodules)	50.5 ± 12.6	0.3:1	Thyroid	Malignant thyroid nodule	Histopathology	Elastic modulous and SWS	3.52 m/s	Aplio500, Toshiba Medical Systems
Duan et al. (65)	China	Prospective cohort	118 (137 lesions)	45.9 ± 13.4	0.6:1	Thyroid	Malignant thyroid nodule	Histopathology	SWE mean	34.5	Aixplorer; Supersonic Imagine
Liu et al. (66)	China	Prospective cohort	238 (254 lesions)	50.9 ± 11.9	0.3:1	Thyroid	Malignant thyroid nodule	Histopathology	SWS	2.78 m/s	N/A
Liu et al. (67)	China	Retrospective cohort	227 (313 lesions)	46.14 ± 9.70	0.2:1	Thyroid	Malignant thyroid nodule	Histopathology	Emax	51.95 Kpa	N/A
Kim et al. (68)	Korea	Retrospective cohort	99 (99 lesions)	45.7+13	N/A	Thyroid	Malignant thyroid nodule	Histopathology	Emean	62 Kpa	Aixplorer US system (SuperSonic Imagine)
Deng et al. (69)	China	Prospective cohort	146 (175 nodules)	46.36 ± 12.5	0.4:1	Thyroid	Malignant thyroid nodule	Histopathology	SWS	2.59 m/s.	Siemens Acuson S2000 US machine
Baig et al. (70)	China	Prospective cohort	122 (163 nodules)	53 ± 13.7	0.2:1	Thyroid	Malignant thyroid nodule	Histopathology	Emax	67.3 Kpa	Aixplorer, Supersonic Imagine
Dobruch-Sobczak et al. (71)	Poland	Prospective cohort	119 (169 lesions)	49.2+14	0.3:1	Thyroid	Characterization of thyroid nodules	Histopathology	Emean	30.5 Kpa	Aixplorer, SuperSonic Imagine
Liu et al. (72)	China	Prospective cohort	49 (64 lesions)	45.3 ± 13.1	0.4:1	Thyroid	benign vs. malignant solid Thyroid lesions	Histopathology	Emean	38.3 Kpa	Q-box TM; Super Sonic Imagine
Park et al. (73)	Korea	Retrospective cohort	453 (476 nodules)	45.7+10.33	0.2:1	Thyroid	Benign vs. malignant solid Thyroid lesions	Histopathology	Emean	85.2 Kpa	Aixplorer, SuperSonic Imagine
Samir et al. (74)	USA	Prospective cohort	35 (35 lesions)	55 + 16.1	0.5:1	Thyroid	Benign vs. malignant thyroid follicular lesions	Histopathology	Young's Modulus	22.3 Kpa	Aixplorer, SuperSonic Imagine
Yang et al. (75)	China	Prospective cohort	107 (107 lesions)	54.0 ± 9.4	0.26:1	Thyroid	Benign vs. malignant solid Thyroid lesions	Histopathology	Mean SWS	3.01 m/s	Acuson S3000 (Siemens)
Zhou et al. (76)	China	Prospective cohort	290 (302 lesions)	49.80+12.34	0.4:1	Thyroid	Benign vs. malignant solid Thyroid lesions	Histopathology	Mean SWS	2.6 m/s	Acuson S3000 (Siemens)

**Table 2:** Baseline characteristics of enrolled patients and criteria of the used CEUS system.

**References**	**Country**	**Study design**	**Organ**	**Condition**	**Patients/ Lesions (N)**	**Age (Years)**	**Male: Female**	**Contrast agent**	**Reference test**	**US technique**	**Mechanical index**	**Probe**
Bertolotto et al. (5)	Italy	Retrospective	Kidney	Indeterminate renal masses with equivocal enhancement on CT	47 (30 HP)	65 ± 13	4.75:1	2.4 mL SonoVue	Histopathology	Pulse inversion harmonic imaging Cadence contrast pulse sequencing	0.05–0.21	Convex array (C5–1) & (4C1) &(C5–2 HDI) & (CA430E)
Cai et al. (77)	China	Prospective cohort	Kidney	Benign vs. malignant renal masses	73 (73 lesions)	56.36 ± 12.2	1.6:1	1.0–1.8 mL SonoVue	Histopathology and follow up data	Acuson Sequoia 512, Siemens,	0.21–0.23	4C1-S convex probe 1–4 MHz
Chang et al. (30)	USA	Prospective cohort	Kidney	Renal solid and cystic lesions	44 (23 HP lesions)	56 ± 14	0.7:1	Sonazoid	Histopathology and follow up data	Siemens Acuson Sequoia 512	0.19	4C1 abdominal transducer
Chen et al. (78, 79)	China	Prospective cohort	Kidney	RCC vs. AML	99 (102 lesions)	56.6 ± 16.5	2:01	1.2 ml of SonoVue	Histopathology	Acuson S2000 (contrast pulse sequencing)	N/A	N/A
Chen et al. (80)	China	Prospective cohort	Kidney	Complex cystic renal masses	59 (71 lesions)	49.6 + 14.25	2.9:1	2.4 mL of SonoVue	Histopathology and follow up data	Coded phase inversion harmonic imaging (Logiq 9 scanner GE Healthcare)	0.07–0.10	3.5C (2.5–5.0 MHz) and 4C (1.0–4.0 MHz) convex transducers
Defortescu et al. (81)	France	Prospective cohort	Kidney	Complex renal cysts	47 (47 lesions)	46 + 9.75	1.8:1	1.2 mL SonoVue	Histopathology and follow up data	ACUSON S2000-Siemens−10	0.06–0.1	Convex probe 3–4.5 mHz
Li et al. (16)	China	Retrospective	Kidney	RCC vs. AML	411 (429 lesions)	54.12 ± 12.57	1.9:1	1.2 mL SonoVue	Histopathology	E9 system (GE Healthcare	0.11	C1-5, 1–5 MHz
Li et al. (82)	China	Retrospective	Kidney	Solid Renal Masses	91 (100 lesions)	62.0 ± 15.6	2.6:1	1.0–1.2 ml SonoVue	Histopathology	Acuson Sequoia 512 scanner	< 0.2	4V1 vector transducer, 1–4 MHz
Lu et al. (83)	China	Retrospective	Kidney	RCC vs. AML	189 (189 lesions)	47.3 ± 20.7	1.6:1	1.2 ml SonoVue	Histopathology	LOGIC E9	< 0.1	C1–5, 1.5 MHz
Nicolau et al. (84)	Spain	Prospective cohort	Kidney	Indeterminate renal masses by CT	72 (83 nodules)	64.9 + 14.5	1.9:1	2.4 mL of SonoVue	Histopathology and follow up data	Cadence contrast pulse sequencing technology (CPS)	< 0.2 at Sequoia 512, < 0.009 at S2000)	4C1 convex array probe
Oh et al. (85)	Korea	Retrospective	Kidney	RCC vs. AML (small masses)	49 lesions	61+11.5	2.5:1	SonoVue	Histopathology	N/A	N/A	N/A
Sanz et al. (86)	Spain	Prospective cohort	Kidney	Complex cystic renal masses	66 (67 lesions)	67.8+ 1.83	2.7:1	2.4 mL SonoVue	Histopathology	Hitachi Preirus	N/A	EUP-C715 probe (5–1 MHz
Tamas-Szora et al. (87)	Romania	Prospective cohort	Kidney	RCC	32 (33 lesions)	60.9 ± 10.43	1:01	1.6 mL of SonoVue	Histopathology	General Electric Logiq 7 system	0.09–0.11	Convex wide-band transducer (2–5.5 MHz)
Tian et al. (28)	China	Prospective cohort	Kidney	Renal SOL	367 (378 lesions)	N/A	N/A	1.2 mL SonoVue	Histopathology	ACUSON S2000 Ultrasound System		Probe 4C1, 2.5–5 MHz
Wei et al. (88)	China	Retrospective	Kidney	Benign vs. malignant solid renal masses	118 (118 lesions)	53.5 ± 12.6	1.6:1	1.6–2.4 mL SonoVue	Histopathology	Contrast pulse sequence, Sequoia 512 ultrasound system (Siemens	0.18−0.20	4C1, 3–4 MHz
Yong et al. (89)	Singapore	Retrospective	Kidney	Undetermined renal masses	63 (74 nodules)	62.4 ± 14.5	1.6:1	1.5 ml of SonoVue	Histopathology	Aplio 500, Toshiba Medical Systems AND iU22, Philips Healthcare	N/A	N/A
Zhang et al. (90)	China	Prospective cohort	Kidney	Benign vs. malignant thyroid nodules	148 (157 lesions)	45.4 ± 10.5	N/A	2.4 ml SonoVue	Histopathology	Contrast pulse sequence (CPS) imaging. Acuson, Sequoia 512 Encompass	0.20–0.23	15L8w probe (8–14 MHz)
Miyamoto et al. (91)	Japan	Prospective cohort	Breast	Focal breast lesions	127 (127 lesions)	48.5 ± 12.3	:1	0.015 mL/kg Sonazoid	Histopathology	AplioXG, Toshiba AND, Hitachi-Aloka AND Logiq E9, GE	0.1–0.4	Broadband linear phased-array transducer
Xia et al. (92)	China	Retrospective	Breast	Papillary breast lesions	50 (52 lesions)	51 +13.57	:1	2.4 mL SonoVue	Histopathology	Pulse-inverse harmonic imaging technique [Philips iU22]	0.05–0.08	3–9-MHz linear transducer
Xiao et al. (93)	China	Prospective cohort	Breast	Subcentimetric breast lesions	203 (209 lesions)	47+15.25	:1	4.8 mL of SonoVue	Histopathology	Pulse inversion harmonic technique w iU22 (Philips)	0.06	9–3-MHz linear transducer
Yuan et al. (94)	China	Prospective cohort	Breast	Breast tumors	216 (216 lesions)	46 ± 12	:1	2.5 mL SonoVue	Histopathology	Sequoia; Siemens Medical Solutions	N/A	10 MHz transducer
Aubé et al. (95)	France	Prospective cohort	Liver	Diagnosis of HCC (< 3 cm)	381 (544 lesions)	62 ± 9.69	4.6:1	SonoVue	Histopathology, CT and MRI according to EASL-AASLD	N/A	N/A	N/A
Beyer et al. (96)	Germany	Retrospective	Liver	Benign vs. malignant liver nodules	83 (83 lesions)	59.8 +10	2.6:1	1–2.4 ml SonoVue	Histopathology	LOGIQ E9, GE	N/A	1–6 MHz curved probe
Corvino et al. (97)	Italy	Prospective cohort	Liver	Cystic and cyst like liver lesions	48 (50 lesions)	65+15	0.9:1	2.4 or 4.8 mL SonoVue	Histopathology	MyLab 70 Twice scanner (Esaote)	N/A	D multifrequency (2.5–5 MHz) convex probes
Feng et al. (98)	China	Retrospective	Liver	HCC differentiation	271 (374 lesions)	49.25 + 17	3.9:1.0	2.4 mL SonoVue	Histopathology	iU22 system (Philips)	< 0.1	(5–2 MHz) convex transducer (C5-2).
Iwamoto et al. (99)	Japan	Retrospective	Liver	Macroscopic HCC	77 (79 lesions)	70 ± 9	2.7:1	0.015 ml/kg Sonazoid	Histopathology	(tissue harmonic grayscale imaging) LOGIQ 7 or E9 US	0.2–0.3	Convex or linear probes with a frequency of 2–5 or 4–9 MHz
Kobayashi et al. (100)	Japan	Retrospective	Liver	NS-HCC	85 (85 lesions)	66 + 13.75	2.9:1	0.015 ml/kg Sonazoid	Histopathology	Wide-band pulse-inversion harmonic imaging (HI VISION Ascendus (Hitachi))	0.16–0.2	Microconvex probe (EUP- C715, 3.5 MHz
Kobayashi et al. (101)	Japan	Retrospective	Liver	Liver metastasis	98 (148 lesions)	66.46 ± 11.2	1.7:1	0.0075 mL/kg Sonazoid	Histopathology	SSA 770 A or 790 A US system (Toshiba)	0.17–0.27	3.75-MHz convex probe
Liu et al. (12)	China	Prospective cohort	Liver	Hyperechoic HFL	102 (135 lesions)	51.4 ± 12.5	2.8:1	1.5 mL of SonoVue	Histopathology	GE Logiq9 color Doppler ultrasonography	0.11	convex array probe (frequency: 3.5–5 MHz)
Quaia et al. (102)	Italy	Retrospective	Liver	Benign vs. malignant liver lesions in cirrhotic patients	46 (55 lesions)	55 ± 10	0.8:1	2.4 mL SonoVue	Histopathology	Sequoia, Acuson-Siemens AND iU22 (iU22; Philip)	0.09–0.14	Convex array 2–4 MHz 4C1 transducer AND 2–5-MHz broadband curvilinear probe
Sandrose et al. (103)	USA	Retrospective	Liver	CT undetermined HFL	78 (163 lesions)	61.8 + 15.25	1.1:1	1.2 ml bolus of SonoVue	Histopathology and PET/CT follow up	Pulse inversion harmonic imaging (GE LOGIQ 9E)	N/A	N/A
Schellhaas et al. (104)	Germany	Prospective cohort	Liver	HCC by CEUS and ESCULAP	100 (100 lesions)	66.1 + 7.17	5.7:1	1.5 ml SonoVue	Histology and imaging	GE Logiq E9 AND Siemens Acuson S2000 AND Toshiba Aplio 500	N/A	N/A
Tada et al. (105)	Japan	Prospective cohort	Liver	Macroscopic HCC	99 (99 lesions)	67.8 ± 10.4	2.7:1	0.015 ml/kg of Sonazoid	Histopathology	Wideband harmonic imaging (Aplio XG system, Toshiba)	(0.18–0.28)	5-MHz convex transducer 1.4 and 5.3 MHz
Thakur et al. (106)	India	Prospective cohort	Liver	HCC	50 (50 lesions)	52 + 14.25	1.4:1	2.4 ml SonoVue	Histopathology, CT and MRI	Xario XG (Toshiba)	< 0.2	
Wang et al. (64)	Germany	Prospective cohort	Liver	Superficial HFL	27 (27 lesions)	N/A	2.4:1	2.4 ml SonoVue	Histopathology, one patient by MRI	Philips iU22, LOGIQ E9, Aplio 500	N/A	High frequency transducer (7.5–12 MHz)
Wu et al. (107)	China	Prospective cohort	Liver	Focal hepatic lesions	46 (55 lesions)	46.5 + 15.2	1.2:1	2.4-mL dose of SonoVue	Histopathology, CECT and MRI	Philips iU22 US system	0.06	5C2 multi- frequency convex probe
Yin et al. (108)	China	Prospective cohort	Liver	Cholangiocarcinoma vs. inflammatory lesions	40 (40 lesions)	58.7 + 9.701	1.4:1	1.5 mL of SonoVue	Histopathology	LOGIQ E9 (GE Healthcare)	< 0.1	C5-1, 2.0–4.0 MHz
Zhang et al. (109)	China	Prospective cohort	Liver	Benign vs. malignant liver lesions	156 (176 lesions)	50.7 + 16.25	1.9:1	2.4 mL of SonoVue	Histopathology	Acuson S2000 ultrasound system Seimens	N/A	4C1 convex array probe; frequency 2.0–4.0 MHz
Takahashi et al. (110)	Japan	Prospective cohort	Liver	HFL < 30 mm	56 (67 lesions)	65.8 ± 10.1	2.5:1	0.0075 mL/kg Sonazoid	Histopathology	SSA-790A ultrasound system (Aplio	(0.20–0.25)	3.75 MHz convex probe
Taimr et al. (111)	Canada	Prospective cohort	Liver	Liver metastasis	89 (89 lesions)	31–87	1.6:1	1.5–2.4 mL SonoVue	Histopathology	Contrast-tuned imaging Hitachi 900 and Hitachi Preirus	N/A	2.5–5.0 MHz probe
Cantisani et al. (9)	Italy	Prospective cohort	Thyroid	Thyroid nodules	48 (53 lesions)	49.4 + 8.75	2.7:1	4.8 mL SonoVue	Histopathology	MyLab 70XvG, Esaote	N/A	Linear probe (7–12 MHz) (N:36)
Deng et al. (69)	China	Prospective cohort	Thyroid	Malignant thyroid nodule	146 (175 nodules)	46.36 ± 12.5	0.4:1	2.4 mL of the SonoVue	Histopathology	Siemens Acuson S2000 US machine	0.1	9L4, 5.0 MHz to 14.0 MHz
Diao et al. (112)	China	Prospective cohort	Thyroid	Benign vs. malignant thyroid nodules	77 (87 lesions)	52.4 ± 17.2	N/A	1.5 mL SonoVue	Histopathology	Siemens Acuson S2000 US	0.06–0.1	5- to 14-MHz linear array transducer (9L4)
Giusti et al. (113)	Italy	Prospective cohort	Thyroid	Benign vs. malignant thyroid nodules	63 (HP in 38 lesions)	55.9 ± 14.7	0.2:1	4.8 ml of SonoVue	Histopathology	MyLab 70 US scanner	N/A	7.5-MHz linear probe
Jiang et al. (114)	China	Prospective cohort	Thyroid	Benign vs. malignant calcified thyroid nodules	122 (122 nodules)	46 + 12	0.4:1	2.4 mL of the SonoVue	Histopathology	Contrast pulse sequencing (CPS) (ACUSON Sequoia 512 (Siemens Healthcare)	0.32	15L8w high- frequency linear transducer
Wu et al. (107)	China	Retrospective	Thyroid	Benign vs. malignant thyroid nodules	133 lesions	46.3 + 10	0.5:1	1.2 mL SonoVue	Histopathology	ESAOTE MyLab 90 X-vision	0.05–0.07)	L522 (3–9 MHz) linear-array probe
Zhang et al. (46)	China	Prospective cohort	Thyroid	Benign vs. malignant thyroid nodules	70 (200 lesions)	49.6 + 12.8	0.3:1	2.0 mL SonoVue	Histopathology	Acuson S2000	< 0.10	9-MHztransducer
Zhang et al. (90)	China	Prospective cohort	Thyroid	Benign vs. malignant thyroid nodules	246 (319 patients)	46.1 ± 15.2	0.5:1	2.4 ml SonoVue	Histopathology	Contrast pulsed sequencing (CPS) Siemens Acuson S2000	N/A	9 L4 transducer
Zhang et al. (90)	China	Prospective cohort	Thyroid	Benign vs. malignant thyroid nodules	111 (145 nodules)	48 + 13.45	0.2:1	1.6 mL SonoVue	Histopathology	Contrast tuned imaging Mylab Twice Esaote	N/A	LA522 transducer (3–9 MHz)
Zhou et al. (115)	China	Prospective cohort	Thyroid	Benign vs. malignant thyroid nodules	161 (167 lesions)	44.14 + 12.01	0.4:1	2.4 ml SonoVue	Histopathology	DC-8EXP; Mindray	0.15	L12-3E transducer

### Outcomes of Pair-Wise Meta-Analysis

#### Breast Cancer

**Figure 2:**
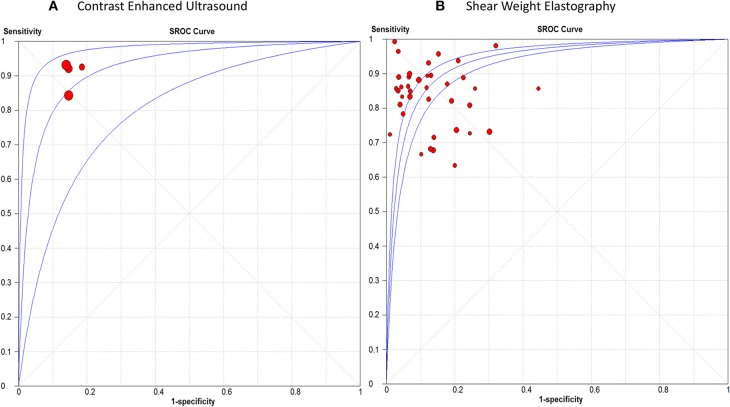
Summary receiver operating characteristic curve of **(A)** Contrast Enhanced Ultrasound, and **(B)** Shear Weight Elastography in breast cancer diagnosis.

Detailed figures for pairwise meta-analysis in all five organs are illustrated in Supplementary File III. The pooled sensitivity, specificity, positive LR, and negative LR for CEUS in detection of breast malignant lesions were 0.89 (95% CI, 0.85, 0.92), 0.85 (95% CI, 0.81, 0.89), 6.13 (95% CI, 4.70, 8.01), and 0.12 (95% CI, 0.07, 0.21), respectively. The pooled DOR was 49.66 (95% CI, 29.42, 83.82) and the area under the receiving-operating characteristic (AUROC) curve was 0.92, Figure 2A. No heterogeneity was observed for sensitivity (*p* = 0.15) or specificity (*p* = 0.95).

For SWE, the pooled sensitivity, specificity, positive LR, and negative LR were 0.84 (95% CI, 0.83, 0.86), 0.86 (95% CI, 0.85, 0.87), 7.12 (95% CI, 5.54, 9.15), and 0.18 (95% CI, 0.15, 0.22), respectively. The pooled DOR was 46.22 (95% CI, 31.33, 68.18) with an AUROC of 0.93, Figure 2B. Significant heterogeneity was observed for sensitivity (*p* < 0.0001) and specificity (*p* < 0.0001).

#### Hepatic Cancer

**Figure 3:**
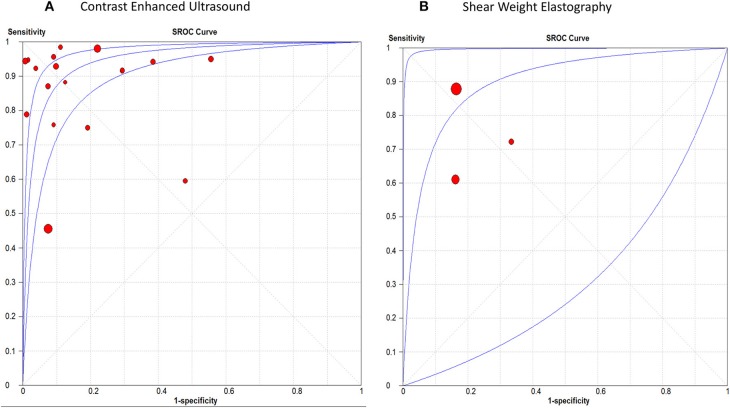
receiver operating characteristic curve of **(A)** Contrast Enhanced Ultrasound, and **(B)** Shear Weight Elastography in hepatic cancer diagnosis.

The pooled sensitivity, specificity, positive LR, and negative LR for CEUS in differentiating malignant hepatic lesions were 0.78 (95% CI, 0.76, 0.81), 0.89 (95% CI, 0.87, 0.91), 6.51 (95% CI, 3.90, 10.85), and 0.13 (95% CI, 0.06, 0.25), respectively. The overall DOR was 57.94 (95% CI, 24.78, 135.45) with an AUROC of 0.95, Figure 3A. The included studies were heterogeneous in the estimates of sensitivity (*p* < 0.0001) and specificity (*p* < 0.0001).

For SWE, the pooled sensitivity, specificity, positive LR, and negative LR were 0.82 (95% CI, 0.77, 0.87), 0.83 (95% CI, 0.76, 0.89), 4.30 (95% CI, 2.85, 6.48), and 0.29 (95% CI, 0.12, 0.71), respectively. The overall DOR was 14.46 (95% CI, 4.09, 51.04) with an AUROC of 0.90, Figure 3B. The included studies were heterogeneous in the estimates of sensitivity (*p* < 0.0009) and specificity (*p* < 0.0001).

#### Thyroid Cancer

**Figure 4:**
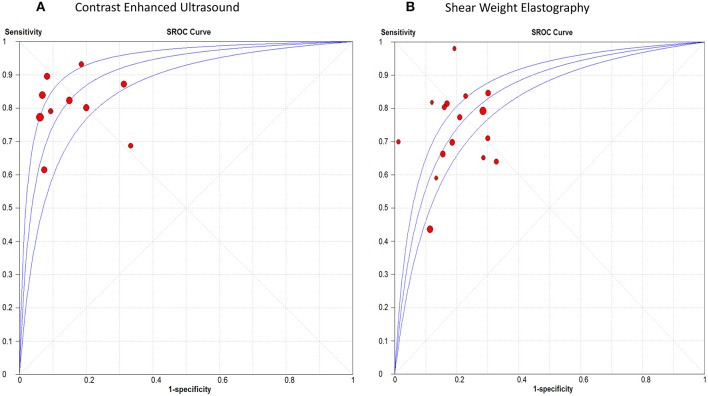
Summary receiver operating characteristic curve of **(A)** Contrast Enhanced Ultrasound, and **(B)** Shear Weight Elastography in thyroid cancer diagnosis.

The pooled sensitivity, specificity, positive LR, and negative LR for CEUS in detecting malignant thyroid nodules were 0.81 (95% CI, 0.78, 0.84), 0.88 (95% CI, 0.86, 0.90), 6.01 (95% CI, 3.88, 9.31), and 0.23 (95% CI, 0.17, 0.31), respectively. The overall DOR was 28.54 (95% CI, 16.79, 48.51) with an AUROC of 0.91, Figure 4A. Significant heterogeneity was observed for sensitivity (*p* = 0.001) and for specificity (*p* < 0.0001).

For SWE, the pooled sensitivity, specificity, positive LR, and negative LR were 0.67 (95% CI, 0.64, 0.69), 0.77 (95% CI, 0.76, 0.79), 3.50 (95% CI, 2.93, 4.18), and 0.33 (95% CI, 0.25, 0.45), respectively. The overall DOR was 11.17 (95% CI, 8.04, 15.51) with an AUROC of 0.84, Figure 4B. Significant heterogeneity was observed for sensitivity (*p* < 0.0001) and specificity (*p* < 0.0001).

#### Renal Cancer

**Figure 5:**
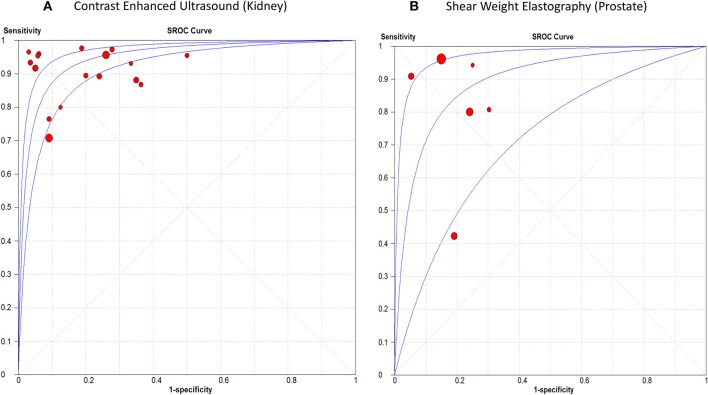
Summary receiver operating characteristic curve of **(A)** Contrast Enhanced Ultrasound in renal cancer diagnosis, and **(B)** Shear Weight Elastography in prostate cancer diagnosis.

The sensitivity of CEUS ranged from 0.71 to 0.98 with a pooled sensitivity of 0.87 (95% CI, 0.85, 0.88). Specificity ranged from 0.50 to 0.97 with a pooled specificity of 0.84 (95% CI, 0.82, 0.87). The pooled positive and negative LRs were 5.55 (95% CI, 3.74, 8.22) and 0.12 (95% CI, 0.07, 0.19), respectively. The overall DOR was 53.44 (95% CI, 29.89, 95.56) with an AUROC of 0.95, Figure 5A. Significant heterogeneity was observed for sensitivity (*p* < 0.0001) and specificity (*p* < 0.0001).

#### Prostate Cancer

The sensitivity of SWE ranged from 0.42 to 0.96 with a pooled sensitivity of 84% (95% CI, 0.80, 0.87). Specificity ranged from 0.70 to 0.95 with a pooled specificity of 0.84 (95% CI, 0.82, 0.86). The pooled positive and negative LRs were 4.59 (95% CI, 2.68, 7.87) and 0.18 (95% CI, 0.07, 0.44), respectively. The overall DOR was 25.35 (95% CI, 7.15, 89.89) with an AUROC of 0.89 (Figure 5A). Significant heterogeneity was observed for sensitivity (*p* < 0.0001) and specificity (*p* < 0.0001) (Figure 5B). Table 3 summarizes the diagnostic results for both tests in different cancer sites.

**Table 3:** Summary of the results of pooled sensitivity, specificity, positive, and negative likelihood ratios for SWE and CEUS in different cancers.

**Cancer**	**Test**	**Sensitivity**	**Specificity**	**+ ve LR**	**-ve LR**	**DOR**	**AUROC**
Breast cancer	SWE	0.84 (95% CI, 0.83, 0.86)	0.86 (95% CI, 0.85, 0.87)	7.12 (95% CI, 5.54, 9.15)	0.18 (95% CI, 0.15, 0.22)	46.22 (95% CI, 31.33, 68.18)	0.93
	CEUS	0.89 (95% CI, 0.85, 0.92)	0.85 (95% CI, 0.81, 0.89)	6.13 (95% CI, 4.70, 8.01)	0.12 (95% CI, 0.07, 0.21)	49.66 (95% CI, 29.42, 83.82)	0.92
Hepatic cancer	SWE	0.82 (95% CI, 0.77, 0.87)	0.83 (95% CI, 0.76, 0.89)	4.30 (95% CI, 2.85, 6.48)	0.29 (95% CI, 0.12, 0.71)	14.46 (95% CI, 4.09, 51.04)	0.90
	CEUS	0.78 (95% CI, 0.76, 0.81)	0.89 (95% CI, 0.87, 0.91)	6.51 (95% CI, 3.90, 10.85)	0.13 (95% CI, 0.06, 0.25)	57.94 (95% CI, 24.78, 135.45)	0.95
Thyroid cancer	SWE	0.67 (95% CI, 0.64, 0.69)	0.77 (95% CI, 0.76, 0.79)	3.50 (95% CI, 2.93, 4.18)	0.33 (95% CI, 0.25, 0.45)	11.17 (95% CI, 8.04, 15.51)	0.84
	CEUS	0.81 (95% CI, 0.78, 0.84)	0.88 (95% CI, 0.86, 0.90)	6.01 (95% CI, 3.88, 9.31)	0.23 (95% CI, 0.17, 0.31)	28.54 (95% CI, 16.79, 48.51)	0.91
Renal carcinoma	CEUS	0.87 (95% CI, 0.85, 0.88)	0.84 (95% CI, 0.82, 0.87)	5.55 (95% CI, 3.74, 8.22)	0.12 (95% CI, 0.07, 0.19)	53.44 (95% CI, 29.89, 95.56)	0.95
Prostate cancer	SWE	84% (95% CI, 0.80, 0.87)	0.84 (95% CI, 0.82, 0.86)	4.59 (95% CI, 2.68, 7.87)	0.18 (95% CI, 0.07, 0.44)	25.35 (95% CI, 7.15, 89.89)	0.89

*AUROC, Area under the receiving-operating curve; CEUS, contrast-enhanced ultrasound; DOR, Diagnostic odds ratio; LR, Likelihood ratio; SWE, Shear wave elastography*.

### Outcomes of Network Meta-Analysis

**Figure 6:**
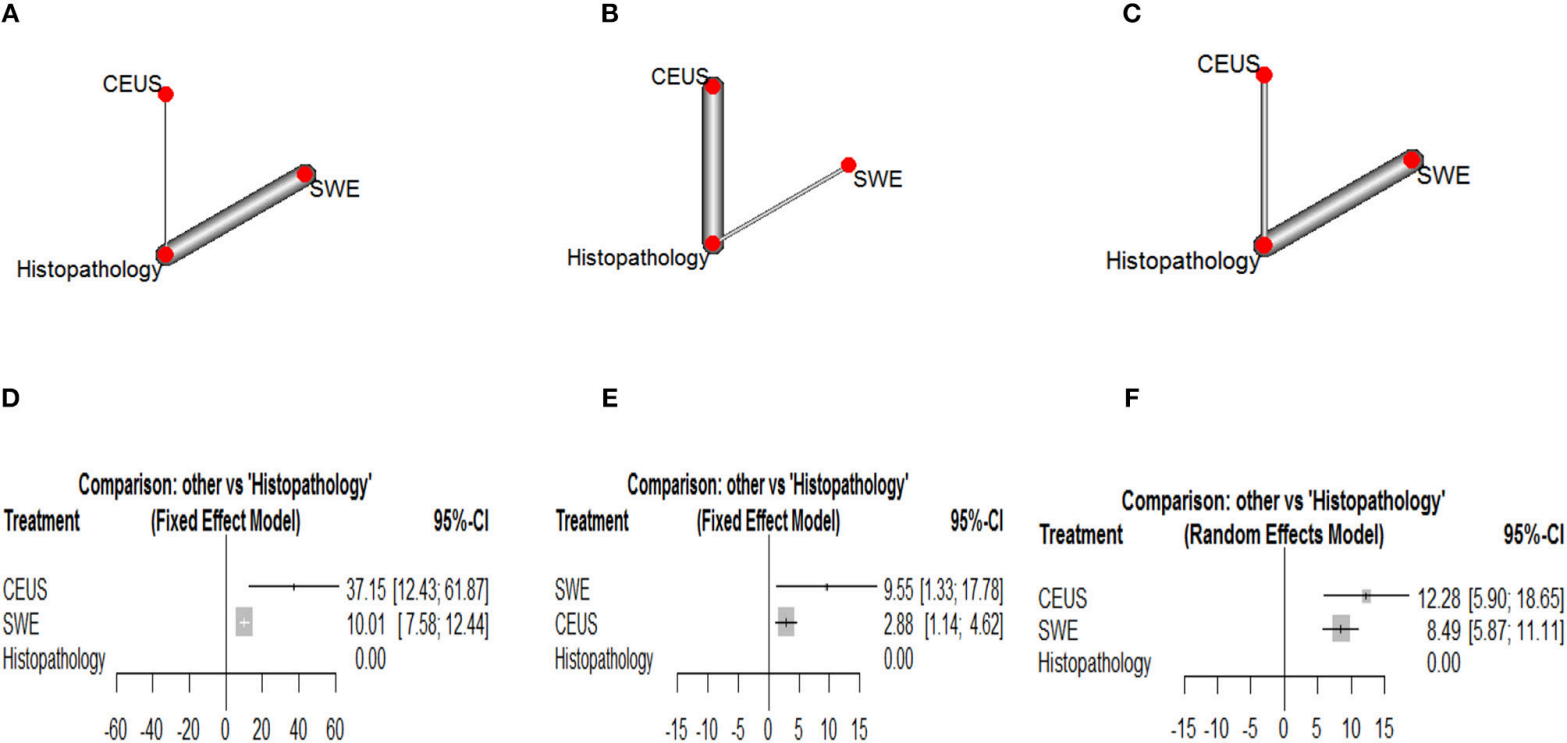
Network plots showing direct evidence between Contrast Enhanced Ultrasound and Shear Weight Elastography in **(A)** breast cancer, **(B)** hepatic caner, and **(C)** thyroid cancer. Also, forest plots of network meta-analysis between Contrast Enhanced Ultrasound and Shear Weight Elastography vs. histopathology in **(A)** breast cancer, **(B)** hepatic caner, and **(C)** thyroid cancer. **(D)** Forest plot CEUS vs. SWE of breast cancer. **(E)** Forest plot CEUS vs. SWE of hepatic cancer. **(F)** Forest plot CEUS vs. SWE of thyroid cancer.

Corresponding network plots and forest plots of network meta-analysis between CEUS and SWE are shown in Figure 6. In breast cancer, NMA showed that CEUS was associated with significantly higher DOR than SWE (DOR = 27.14, 95% CI [2.30, 51.97], *p* = 0.011). While NMA showed no significant difference between CEUS and SWE in detecting hepatic (DOR = −6.67, 95% CI [-15.08, 1.74, *p* = 0.61]) and thyroid malignant lesions (DOR = 3.79, 95% CI [−3.10, 10.68], *p* = 0.58). No significant heterogeneity or inconsistency were observed between the pooled studies for breast (*I*^2^ = 10%, *p* = 0.30) and hepatic cancer (*I*^2^ = 20%, *p* = 0.21). While a *p*-value of 0.05 indicated significant heterogeneity among the studies of thyroid cancer; therefore, the random-effects model was employed.

### Ranking Diagnostic Tests

According to Glas et al. (116), the DOR is considered as an indicator of ranking of competing diagnostic tests. According to our results, CEUS achieved the highest DOR in detecting breast and thyroid malignant lesions, while SWE achieved the highest DOR in detecting hepatic malignant lesions.

## Discussion

This meta-analysis of DTA studies provides a comprehensive assessment and comparison of the diagnostic accuracy of two US modalities in differentiating malignant tumors in different body organs. It showed relatively high sensitivity (between 78 and 89%) and specificity (between 84 and 89%) for CEUS in identifying malignant lesions in the breast, liver, thyroid and kidneys. Moreover, it demonstrated relatively high sensitivity (between 82 and 84%) and specificity (between 83 and 86%) for SWE in differentiating malignant tumors within the breast, liver and prostate. However, it had relatively lower sensitivity (67%) and specificity (77%) in identifying malignant nodules within the thyroid gland.

Our results support some recent practice guidelines that endorse the use of CEUS and SWE in differentiating malignant lesions within the liver and the breast (117, 118). Moreover, it provides new data on a comparison that can impact the clinical practice. Through NMA, we compared the diagnostic accuracy of CEUS and SWE in three organs (where data on both tests were available in the literature). Our network and ranking analysis showed that CEUS was more accurate than SWE in differentiating breast and thyroid lesions (although the difference was not significant in thyroid malignancy according to NMA). On the other hand, SWE ranked higher in terms of diagnostic accuracy in differentiating hepatic malignant lesions (although the difference was not significant according to NMA).

Our results are in agreement with a former meta-analysis by Sadigh et al. that showed high sensitivity and specificity for SWE in differentiating breast malignant lesions [88 and 83% in comparison to 84 and 86% in our analysis; (11)]. However, our sensitivity and specificity results are quite lower than those obtained by Liu et al. in a meta-analysis on SWE accuracy in differentiating thyroid malignancy [sensitivity 81% and specificity 84%; (12)]. Likewise, another meta-analysis reported high sensitivity and specificity (93 and 90%, respectively) for CEUS in identifying hepatic malignant lesions (119). The observed discrepancy between our findings and those of the aforementioned meta-analyses may be attributed to the different sample size (being larger in our analysis) or the lesional characteristics of enrolled patients (being easier to identify in the studies included in the other meta-analysis i.e., less depth and clear contrast from the surrounding tissue).

Interestingly, a meta-analysis by Guang et al. showed comparable diagnostic accuracy for SonoVue-enhanced US with contrast-enhanced computed tomography and magnetic resonance imaging (8). Moreover, CEUS has other advantages over these modalities as ease of access, lack of radiation exposure or nephrotoxic materials; limitations that affect the use of CT and MRI in several diagnostic applications (120, 121). It is also fair to recognize that both tests have limitations as well. For example, SWE suffers from operator-dependency and manual compression, while the adverse effects of the contrast agent is a concern with CEUS use. Further technical improvements with both modalities would further enhance their clinical potential.

### Strength Points

This NMA directly compares the diagnostic accuracies of CEUS and SWE in different cancer sites and using different analytic approaches as pairwise, network and ranking pooled analyses. Therefore, it provides a holistic evaluation of the comparison of both techniques in different body organs. We performed a thorough literature search and retrieved a large number of studies (relatively large sample size), which adds to the validity and generalizability of our findings. Unlike former reviews that retrieved a small number of studies and focused on one test in one organ, we aimed to provide a comprehensive assessment of both tests in different organs and a high quality comparison whenever suitable data were provided.

### Limitations and Future Research Implications

Our meta-analysis has some limitations. First, the observed heterogeneity in the majority of our outcomes may be due to differences in study design and patient characteristics. Second, we could not examine the effects of lesion characteristics, such as size and depth on the diagnostic accuracy of both tests due to lack of data. Third, many of the included studies did not mention whether the results of CEUS or SWE were interpreted with blinding to the findings of histopathology or not. Future studies should report diagnostic accuracy data based on the size and depth of the lesions to allow more detailed analysis. Moreover, they should adhere to the Standards for Reporting of Diagnostic Accuracy “STRAD” checklist in reporting their methods and findings to allow a more thorough critical appraisal.

## Conclusion

Both diagnostic tests (CEUS and SWE) showed relatively high sensitivity and specificity in detecting malignant tumors in different organs; CEUS had higher diagnostic accuracy than SWE in detecting breast and thyroid cancer, while SWE had higher accuracy in detecting hepatic cancer (the differences in the latter two cancer types were not statistically significant). These results endorse the use of both tests for malignancy detection and rank their accuracy in different organs. Future studies should provide more data to allow characterization of both tests in lesions of different size or depth.

## Author Contributions

YS developed the concept, designed the study, and prepared the manuscript. RH acquired the data, controlled quality of the work, analyzed the data, and prepared the manuscript. LJ acquired the data. YX analyzed the data. YG acquired the data. HR acquired the data and conducted the analysis. ZW analyzed the data and prepared the manuscript.

### Conflict of Interest Statement

The authors declare that the research was conducted in the absence of any commercial or financial relationships that could be construed as a potential conflict of interest.

## Supplementary Material

Supplementary File I PRISMA checklist for systematic reviews/meta-analysis.

Supplementary File II Bibliographic Information of Included Studies.

Supplementary File III Additional Pairwise Meta-analysis Figures.
