# Supplementary material for: Comparative Diagnostic Accuracy of Contrast-Enhanced Ultrasound and Shear Wave Elastography in Differentiating Benign and Malignant Lesions: A Network Meta-Analysis
Source: Front Oncol. 2019 Mar 5;9:102. doi: 10.3389/fonc.2019.00102 (PMC6412152; doi:10.3389/fonc.2019.00102)
Supplement: Supplementary File II — Bibliographic Information of Included Studies. [file Table_2.DOCX]

**Supplementary File II: Bibliographic Information of Included Studies**

One-hundred and fourteen studies were eligible for inclusion in our systematic reviews, including 65 studies on shear wave elastography [^1-65^](#_ENREF_1) and 50 studies on contrast-enhanced ultrasound.[^59^](#_ENREF_59)^,^[^66-114^](#_ENREF_66) One study by Deng e al. assessed both modalities.[^59^](#_ENREF_59)

1. Au FW-F, Ghai S, Moshonov H, Kahn H, Brennan C, Dua H *et al.* Diagnostic performance of quantitative shear wave elastography in the evaluation of solid breast masses: determination of the most discriminatory parameter. *American Journal of Roentgenology* 2014; **203**(3): W328-W336.

2. Chang JM, Won J-K, Lee K-B, Park IA, Yi A, Moon WK. Comparison of shear-wave and strain ultrasound elastography in the differentiation of benign and malignant breast lesions. *American Journal of Roentgenology* 2013; **201**(2): W347-W356.

3. Chang JY, Moon JH, Koh SH, Park S-Y, Lee KS. Clinical Application of Shear Wave Elastography in Breast Masses. *Iranian Journal of Radiology* 2017; **14**(1).

4. Choi H, Sohn Y-M, Seo M. Comparison of 3D and 2D shear-wave elastography for differentiating benign and malignant breast masses: focus on the diagnostic performance. *Clinical radiology* 2017; **72**(10): 878-886.

5. Choi HJ, Ko KH, Jung HK. Shear Wave Elastography for Surgically Verified Breast Papillary Lesions: Is It Effective for Differentiation Between Benign and Malignant Lesions? *Journal of Ultrasound in Medicine* 2017; **36**(10): 2007-2014.

6. Choi JS, Han B-K, Ko EY, Ko ES, Shin JH, Kim GR. Additional diagnostic value of shear-wave elastography and color Doppler US for evaluation of breast non-mass lesions detected at B-mode US. *European radiology* 2016; **26**(10): 3542-3549.

7. Chung J, Lee WK, Cha E-S, Lee JE, Kim JH, Ryu YH. Shear-Wave Elastography for the Differential Diagnosis of Breast Papillary Lesions. *PloS one* 2016; **11**(11): e0167118.

8. Dobruch-Sobczak K, Nowicki A. Role of shear wave sonoelastography in differentiation between focal breast lesions. *Ultrasound in Medicine and Biology* 2015; **41**(2): 366-374.

9. Elmoneam GA, Almolla RM, Ahmed AF, Al Ekrashy MA. Supersonic shear waves quantitative elastography and kinetic magnetic resonance dynamic curve in discriminating BI-RADS 4 breast masses: A comparative study. *The Egyptian Journal of Radiology and Nuclear Medicine* 2016; **47**(4): 1773-1782.

10. Golatta M, Schweitzer-Martin M, Harcos A, Schott S, Gomez C, Stieber A *et al.* Evaluation of virtual touch tissue imaging quantification, a new shear wave velocity imaging method, for breast lesion assessment by ultrasound. *BioMed research international* 2014; **2014**.

11. Guo X, Liu Y, Li W. Diagnostic accuracy of shear wave elastography for prediction of breast malignancy in patients with pathological nipple discharge. *BMJ open* 2016; **6**(1): e008848.

12. Hong S, Woo OH, Shin HS, Hwang S-Y, Cho KR, Seo BK. Reproducibility and diagnostic performance of shear wave elastography in evaluating breast solid mass. *Clinical imaging* 2017; **44**: 42-45.

13. Kim GR, Choi JS, Han B-K, Ko EY, Ko ES, Hahn SY. Combination of shear-wave elastography and color Doppler: Feasible method to avoid unnecessary breast excision of fibroepithelial lesions diagnosed by core needle biopsy. *PloS one* 2017; **12**(5): e0175380.

14. Kim SJ, Ko KH, Jung HK, Kim H. Shear wave elastography: is it a valuable additive method to conventional ultrasound for the diagnosis of small (≤ 2 cm) breast cancer? *Medicine* 2015; **94**(42).

15. Klotz T, Boussion V, Kwiatkowski F, Dieu-de Fraissinette V, Bailly-Glatre A, Lemery S *et al.* Shear wave elastography contribution in ultrasound diagnosis management of breast lesions. *Diagnostic and interventional imaging* 2014; **95**(9): 813-824; e-pub ahead of print 2014/06/17; doi 10.1016/j.diii.2014.04.015.

16. Ko KH, Jung HK, Kim SJ, Kim H, Yoon JH. Potential role of shear-wave ultrasound elastography for the differential diagnosis of breast non-mass lesions: preliminary report. *European radiology* 2014; **24**(2): 305-311.

17. Lee BE, Chung J, Cha ES, Lee JE, Kim JH. Role of shear-wave elastography (SWE) in complex cystic and solid breast lesions in comparison with conventional ultrasound. *European journal of radiology* 2015; **84**(7): 1236-1241; e-pub ahead of print 2015/05/06; doi 10.1016/j.ejrad.2015.04.005.

18. Lee EJ, Jung HK, Ko KH, Lee JT, Yoon JH. Diagnostic performances of shear wave elastography: which parameter to use in differential diagnosis of solid breast masses? *European radiology* 2013; **23**(7): 1803-1811.

19. Lee SH, Chang JM, Kim WH, Bae MS, Cho N, Yi A *et al.* Differentiation of benign from malignant solid breast masses: comparison of two-dimensional and three-dimensional shear-wave elastography. *European radiology* 2013; **23**(4): 1015-1026.

20. Li D-D, Xu H-X, Guo L-H, Bo X-W, Li X-L, Wu R *et al.* Combination of two-dimensional shear wave elastography with ultrasound breast imaging reporting and data system in the diagnosis of breast lesions: a new method to increase the diagnostic performance. *European radiology* 2016; **26**(9): 3290-3300.

21. Ng WL, Rahmat K, Fadzli F, Rozalli FI, Mohd-Shah MN, Chandran PA *et al.* Shearwave elastography increases diagnostic accuracy in characterization of breast lesions. *Medicine* 2016; **95**(12).

22. Olgun DÇ, Korkmazer B, Kılıç F, Dikici AS, Velidedeoğlu M, Aydoğan F *et al.* Use of shear wave elastography to differentiate benign and malignant breast lesions. *Diagnostic and Interventional Radiology* 2014; **20**(3): 239.

23. Seo M, Ahn HS, Park SH, Lee JB, Choi BI, Sohn YM *et al.* Comparison and Combination of Strain and Shear Wave Elastography of Breast Masses for Differentiation of Benign and Malignant Lesions by Quantitative Assessment: Preliminary Study. *Journal of Ultrasound in Medicine* 2018; **37**(1): 99-109.

24. Shi XQ, Li JL, Wan WB, Huang Y. A set of shear wave elastography quantitative parameters combined with ultrasound BI-RADS to assess benign and malignant breast lesions. *Ultrasound in Medicine and Biology* 2015; **41**(4): 960-966.

25. Sim Y, Vinnicombe S, Whelehan P, Thomson K, Evans A. Value of shear-wave elastography in the diagnosis of symptomatic invasive lobular breast cancer. *Clinical radiology* 2015; **70**(6): 604-609.

26. Tang L, Xu H-X, Bo X-W, Liu B-J, Li X-L, Wu R *et al.* A novel two-dimensional quantitative shear wave elastography for differentiating malignant from benign breast lesions. *International journal of clinical and experimental medicine* 2015; **8**(7): 10920.

27. Tian J, Liu Q, Wang X, Xing P, Yang Z, Wu C. Application of 3D and 2D quantitative shear wave elastography (SWE) to differentiate between benign and malignant breast masses. *Scientific reports* 2017; **7**: 41216.

28. Yang Y-P, Xu X-H, Guo L-H, He Y-P, Wang D, Liu B-J *et al.* Qualitative and quantitative analysis with a novel shear wave speed imaging for differential diagnosis of breast lesions. *Scientific reports* 2017; **7**: 40964.

29. Youk JH, Gweon HM, Son EJ, Han KH, Kim J-A. Diagnostic value of commercially available shear-wave elastography for breast cancers: integration into BI-RADS classification with subcategories of category 4. *European radiology* 2013; **23**(10): 2695-2704.

30. Youk JH, Son EJ, Park AY, Kim J-A. Shear-wave elastography for breast masses: local shear wave speed (m/sec) versus Young modulus (kPa). *Ultrasonography* 2014; **33**(1): 34.

31. Li X-L, Xu H-X, Bo X-W, Liu B-J, Huang X, Li D-D *et al.* Value of Virtual Touch Tissue Imaging Quantification for Evaluation of Ultrasound Breast Imaging-Reporting and Data System Category 4 Lesions. *Ultrasound in Medicine and Biology* 2016; **42**(9): 2050-2057.

32. Liu H, Zhao L-X, Xu G, Yao M-H, Zhang A-H, Xu H-X *et al.* Diagnostic value of virtual touch tissue imaging quantification for benign and malignant breast lesions with different sizes. *International journal of clinical and experimental medicine* 2015; **8**(8): 13118.

33. Yao M, Wu J, Zou L, Xu G, Xie J, Wu R *et al.* Diagnostic value of virtual touch tissue quantification for breast lesions with different size. *BioMed research international* 2014; **2014**.

34. Wu S, Cui X, Huang L, Bai X. Combining Virtual Touch Tissue Imaging and BI-RADS May Improve Solid Breast Lesion Evaluation. *Breast Care* 2017; **12**(2): 97-100.

35. Youk JH, Son EJ, Gweon HM, Kim H, Park YJ, Kim J-A. Comparison of strain and shear wave elastography for the differentiation of benign from malignant breast lesions, combined with B-mode ultrasonography: qualitative and quantitative assessments. *Ultrasound in Medicine and Biology* 2014; **40**(10): 2336-2344.

36. Zhang S-P, Zeng Z, Liu H, Yao M-H, Xu G, Wu R. Combination of conventional ultrasonography and virtual touch tissue imaging quantification for differential diagnosis of breast lesions smaller than 10 mm. *Clinical hemorheology and microcirculation* 2017; **67**(1): 59-68.

37. Cong R, Li J, Wang X. Comparing Performance of Combinations of Shear Wave Elastography and B-Mode Ultrasound in Diagnosing Breast Masses: Is It Influenced by Mass Size? *Ultrasound in Medicine and Biology* 2017; **43**(10): 2133-2143.

38. Park J, Woo OH, Shin HS, Cho KR, Seo BK, Kang EY. Diagnostic performance and color overlay pattern in shear wave elastography (SWE) for palpable breast mass. *European journal of radiology* 2015; **84**(10): 1943-1948.

39. Wang ZL, Li Y, Wan WB, Li N, Tang J. Shear-Wave Elastography: Could it be Helpful for the Diagnosis of Non-Mass-Like Breast Lesions? *Ultrasound in Medicine and Biology* 2017; **43**(1): 83-90.

40. Kasai Y, Moriyasu F, Saito K, Hara T, Kobayashi Y, Nakamura I *et al.* Value of shear wave elastography for predicting hepatocellular carcinoma and esophagogastric varices in patients with chronic liver disease. *Journal of Medical Ultrasonics* 2015; **42**(3): 349-355.

41. Gerber L, Fitting D, Srikantharajah K, Weiler N, Kyriakidou G, Bojunga J *et al.* Evaluation of 2D-Shear Wave Elastography for Characterisation of Focal Liver Lesions. *Journal of Gastrointestinal & Liver Diseases* 2017; **26**(3).

42. Özmen E, Adaletli İ, Kayadibi Y, Emre Ş, Kılıç F, Dervişoğlu S *et al.* The impact of share wave elastography in differentiation of hepatic hemangioma from malignant liver tumors in pediatric population. *European journal of radiology* 2014; **83**(9): 1691-1697.

43. Tian W-S, Lin M-X, Zhou L-Y, Pan F-S, Huang G-L, Wang W *et al.* Maximum value measured by 2-D shear wave elastography helps in differentiating malignancy from benign focal liver lesions. *Ultrasound in Medicine and Biology* 2016; **42**(9): 2156-2166.

44. Ahmad S, Cao R, Varghese T, Bidaut L, Nabi G. Transrectal quantitative shear wave elastography in the detection and characterisation of prostate cancer. *Surgical endoscopy* 2013; **27**(9): 3280-3287.

45. Boehm K, Salomon G, Beyer B, Schiffmann J, Simonis K, Graefen M *et al.* Shear wave elastography for localization of prostate cancer lesions and assessment of elasticity thresholds: implications for targeted biopsies and active surveillance protocols. *The Journal of urology* 2015; **193**(3): 794-800.

46. Porsch M, Wendler JJ, Liehr U-B, Lux A, Schostak M, Pech M. New aspects in shear-wave elastography of prostate cancer. *Journal of ultrasonography* 2015; **15**(60): 5.

47. Woo S, Kim SY, Cho JY, Kim SH. Shear wave elastography for detection of prostate cancer: a preliminary study. *Korean journal of radiology* 2014; **15**(3): 346-355.

48. Correas JM, Tissier AM, Khairoune A, Vassiliu V, Mejean A, Helenon O *et al.* Prostate cancer: diagnostic performance of real-time shear-wave elastography. *Radiology* 2015; **275**(1): 280-289; e-pub ahead of print 2015/01/20; doi 10.1148/radiol.14140567.

49. Glybochko P, Alyaev Y, Amosov A, Krupinov G, Ganzha T, Vorobev A *et al.* Prostate cancer detection by assessing stiffness of different tissues using shear wave ultrasound elastog-raphy. *Urologiia (Moscow, Russia: 1999)* 2016(3): 56-61.

50. Zhang H, Shi Q, Gu J, Jiang L, Bai M, Liu L *et al.* Combined value of Virtual Touch tissue quantification and conventional sonographic features for differentiating benign and malignant thyroid nodules smaller than 10 mm. *Journal of Ultrasound in Medicine* 2014; **33**(2): 257-264.

51. Azizi G, Keller JM, Mayo ML, Piper K, Puett D, Earp KM *et al.* Thyroid nodules and shear wave elastography: a new tool in thyroid cancer detection. *Ultrasound in Medicine and Biology* 2015; **41**(11): 2855-2865.

52. Liu B-X, Xie X-Y, Liang J-Y, Zheng Y-L, Huang G-L, Zhou L-Y *et al.* Shear wave elastography versus real-time elastography on evaluation thyroid nodules: a preliminary study. *European journal of radiology* 2014; **83**(7): 1135-1143.

53. Liu B, Liang J, Zheng Y, Xie X, Huang G, Zhou L *et al.* Two-dimensional shear wave elastography as promising diagnostic tool for predicting malignant thyroid nodules: a prospective single-centre experience. *European radiology* 2015; **25**(3): 624-634.

54. Liu Z, Jing H, Han X, Shao H, Sun Y-x, Wang Q-c *et al.* Shear wave elastography combined with the thyroid imaging reporting and data system for malignancy risk stratification in thyroid nodules. *Oncotarget* 2017; **8**(26): 43406.

55. Liu B-J, Zhao C-K, Xu H-X, Zhang Y-F, Xu J-M, Li D-D *et al.* Quality measurement on shear wave speed imaging: diagnostic value in differentiation of thyroid malignancy and the associated factors. *Oncotarget* 2017; **8**(3): 4948.

56. Wang D, He Y-P, Zhang Y-F, Liu B-J, Zhao C-K, Fu H-J *et al.* The diagnostic performance of shear wave speed (SWS) imaging for thyroid nodules with elasticity modulus and SWS measurement. *Oncotarget* 2017; **8**(8): 13387.

57. Duan S-B, Yu J, Li X, Han Z-Y, Zhai H-Y, Liang P. Diagnostic value of two-dimensional shear wave elastography in papillary thyroid microcarcinoma. *OncoTargets and therapy* 2016; **9**: 1311.

58. Kim H, Kim J-A, Son EJ, Youk JH. Quantitative assessment of shear-wave ultrasound elastography in thyroid nodules: diagnostic performance for predicting malignancy. *European radiology* 2013; **23**(9): 2532-2537.

59. Deng J, Zhou P, Tian S-m, Zhang L, Qian Y. Comparison of diagnostic efficacy of contrast-enhanced ultrasound, acoustic radiation force impulse imaging, and their combined use in differentiating focal solid thyroid nodules. *PLoS One* 2014; **9**(3): e90674.

60. Baig FN, Liu SY, Lam H-C, Yip S-P, Law HK, Ying M. Shear Wave Elastography Combining with Conventional Grey Scale Ultrasound Improves the Diagnostic Accuracy in Differentiating Benign and Malignant Thyroid Nodules. *Applied Sciences* 2017; **7**(11): 1103.

61. Dobruch-Sobczak K, Zalewska EB, Gumińska A, Słapa RZ, Mlosek K, Wareluk P *et al.* Diagnostic performance of shear wave elastography parameters alone and in combination with conventional B-mode ultrasound parameters for the characterization of thyroid nodules: a prospective, dual-center study. *Ultrasound in Medicine and Biology* 2016; **42**(12): 2803-2811.

62. Park AY, Son EJ, Han K, Youk JH, Kim J-A, Park CS. Shear wave elastography of thyroid nodules for the prediction of malignancy in a large scale study. *European journal of radiology* 2015; **84**(3): 407-412.

63. Samir AE, Dhyani M, Anvari A, Prescott J, Halpern EF, Faquin WC *et al.* Shear-wave elastography for the preoperative risk stratification of follicular-patterned lesions of the thyroid: diagnostic accuracy and optimal measurement plane. *Radiology* 2015; **277**(2): 565-573.

64. Yang Y-P, Xu X-H, Bo X-W, Liu B-J, Guo L-H, Xu J-M *et al.* Comparison of virtual touch tissue imaging & quantification (VTIQ) and Virtual touch tissue quantification (VTQ) for diagnosis of thyroid nodules. *Clinical hemorheology and microcirculation* 2017; **65**(2): 137-149.

65. Zhou H, Zhou XL, Xu HX, Li DD, Liu BJ, Zhang YF *et al.* Virtual Touch tissue imaging and quantification in the evaluation of thyroid nodules. *Journal of Ultrasound in Medicine* 2017; **36**(2): 251-260.

66. Bertolotto M, Cicero C, Perrone R, Degrassi F, Cacciato F, Cova MA. Renal masses with equivocal enhancement at CT: characterization with contrast-enhanced ultrasound. *American Journal of Roentgenology* 2015; **204**(5): W557-W565.

67. Cai Y, Du L, Li F, Gu J, Bai M. Quantification of enhancement of renal parenchymal masses with contrast-enhanced ultrasound. *Ultrasound in Medicine and Biology* 2014; **40**(7): 1387-1393.

68. Chang EH, Chong WK, Kasoji SK, Fielding JR, Altun E, Mullin LB *et al.* Diagnostic accuracy of contrast-enhanced ultrasound for characterization of kidney lesions in patients with and without chronic kidney disease. *BMC nephrology* 2017; **18**(1): 266.

69. Chen L, Wang L, Diao X, Qian W, Fang L, Pang Y *et al.* The diagnostic value of contrast-enhanced ultrasound in differentiating small renal carcinoma and angiomyolipoma. *Bioscience trends* 2015; **9**(4): 252-258.

70. Chen Y, Wu N, Xue T, Hao Y, Dai J. Comparison of contrast‐enhanced sonography with MRI in the diagnosis of complex cystic renal masses. *Journal of Clinical Ultrasound* 2015; **43**(4): 203-209.

71. Defortescu G, Cornu JN, Béjar S, Giwerc A, Gobet F, Werquin C *et al.* Diagnostic performance of contrast‐enhanced ultrasonography and magnetic resonance imaging for the assessment of complex renal cysts: A prospective study. *International Journal of Urology* 2017; **24**(3): 184-189.

72. Li C-x, Lu Q, Huang B-j, Xue L-y, Yan L-x, Zheng F-y *et al.* Quantitative evaluation of contrast-enhanced ultrasound for differentiation of renal cell carcinoma subtypes and angiomyolipoma. *European journal of radiology* 2016; **85**(4): 795-802.

73. Li X, Liang P, Guo M, Yu J, Yu X, Cheng Z *et al.* Real-time contrast-enhanced ultrasound in diagnosis of solid renal lesions. *Discovery medicine* 2013; **16**(86): 15-25.

74. Lu Q, Xue L-y, Huang B-j, Wang W-p, Li C-x. Histotype differentiation of hypo-echoic renal tumors on CEUS: usefulness of enhancement homogeneity and intensity. *Abdominal imaging* 2015; **40**(6): 1675-1683.

75. Nicolau C, Buñesch L, Paño B, Salvador R, Ribal MJ, Mallofré C *et al.* Prospective evaluation of CT indeterminate renal masses using US and contrast-enhanced ultrasound. *Abdominal imaging* 2015; **40**(3): 542-551.

76. Oh TH, Lee YH, Seo IY. Diagnostic efficacy of contrast-enhanced ultrasound for small renal masses. *Korean journal of urology* 2014; **55**(9): 587-592.

77. Sanz E, Hevia V, Gómez V, Álvarez S, Fabuel J-J, Martínez L *et al.* Renal complex cystic masses: usefulness of contrast-enhanced ultrasound (CEUS) in their assessment and its agreement with computed tomography. *Current urology reports* 2016; **17**(12): 89.

78. Tamas-Szora A, Socaciu M, Crisan N, Dobrota F, Prundus P, Bungardean C *et al.* Investigation of renal cell carcinoma by contrast-enhanced ultrasound-predictive value of time intensity curve analysis in establishing local tumor invasion and stage: a pilot study. *Urology journal* 2015; **12**(3): 2173-2181.

79. Tian W, Lu J, Jiao D, Cong Z. an evaluation of the clinical diagnostic value of contrast-enhanced ultrasound combined with contrast-enhanced computed tomography in space-occupying lesions of the kidney. *OncoTargets and therapy* 2017; **10**: 3493.

80. Wei S-P, Xu C-L, Zhang Q, Zhang Q-R, Zhao Y-E, Huang P-F *et al.* Contrast-enhanced ultrasound for differentiating benign from malignant solid small renal masses: comparison with contrast-enhanced CT. *Abdominal Radiology* 2017; **42**(8): 2135-2145.

81. Yong C, Teo Y-M, Jeevesh K. Diagnostic performance of contrastenhanced ultrasound in the evaluation of renal masses in patients with renal impairment. *Med J Malaysia* 2016; **71**(4): 193-198.

82. Zhang Y, Luo Y-k, Zhang M-b, Li J, Li J, Tang J. Diagnostic accuracy of contrast-enhanced ultrasound enhancement patterns for thyroid nodules. *Medical science monitor: international medical journal of experimental and clinical research* 2016; **22**: 4755.

83. Miyamoto Y, Ito T, Takada E, Omoto K, Hirai T, Moriyasu F. Efficacy of sonazoid (perflubutane) for contrast-enhanced ultrasound in the differentiation of focal breast lesions: phase 3 multicenter clinical trial. *American Journal of Roentgenology* 2014; **202**(4): W400-W407.

84. Xia H-S, Wang X, Ding H, Wen J-X, Fan P-L, Wang W-P. Papillary breast lesions on contrast-enhanced ultrasound: morphological enhancement patterns and diagnostic strategy. *European radiology* 2014; **24**(12): 3178-3190.

85. Xiao X, Jiang Q, Wu H, Guan X, Qin W, Luo B. Diagnosis of sub-centimetre breast lesions: combining BI-RADS-US with strain elastography and contrast-enhanced ultrasound—a preliminary study in China. *European radiology* 2017; **27**(6): 2443-2450.

86. Yuan Z, Quan J, Yunxiao Z, Jian C, Zhu H, Liping G. Diagnostic value of contrast-enhanced ultrasound parametric imaging in breast tumors. *Journal of breast cancer* 2013; **16**(2): 208-213.

87. Aubé C, Oberti F, Lonjon J, Pageaux G, Seror O, N'kontchou G *et al.* EASL and AASLD recommendations for the diagnosis of HCC to the test of daily practice. *Liver International* 2017; **37**(10): 1515-1525.

88. Beyer LP, Wassermann F, Pregler B, Michalik K, Rennert J, Wiesinger I *et al.* Characterization of focal liver lesions using CEUS and MRI with liver-specific contrast media: experience of a single radiologic center. *Ultraschall in der Medizin-European Journal of Ultrasound* 2017; **38**(06): 619-625.

89. Corvino A, Catalano O, Corvino F, Sandomenico F, Petrillo A. Diagnostic Performance and Confidence of Contrast-Enhanced Ultrasound in the Differential Diagnosis of Cystic and Cysticlike Liver Lesions. *American Journal of Roentgenology* 2017; **209**(3): W119-W127.

90. Feng Y, Qin X-C, Luo Y, Li Y-Z, Zhou X. Efficacy of contrast-enhanced ultrasound washout rate in predicting hepatocellular carcinoma differentiation. *Ultrasound in Medicine and Biology* 2015; **41**(6): 1553-1560.

91. Iwamoto T, Imai Y, Kogita S, Igura T, Sawai Y, Fukuda K *et al.* Comparison of contrast-enhanced ultrasound and gadolinium-ethoxybenzyl-diethylenetriamine pentaacetic acid-enhanced MRI for the diagnosis of macroscopic type of hepatocellular carcinoma. *Digestive Diseases* 2016; **34**(6): 679-686.

92. Kobayashi T, Aikata H, Hatooka M, Morio K, Morio R, Kan H *et al.* Usefulness of combining gadolinium-ethoxybenzyl-diethylenetriamine pentaacetic acid-enhanced magnetic resonance imaging and contrast-enhanced ultrasound for diagnosing the macroscopic classification of small hepatocellular carcinoma. *European radiology* 2015; **25**(11): 3272-3281.

93. Kobayashi K, Maruyama H, Kiyono S, Yokosuka O, Ohtsuka M, Miyazaki M *et al.* Histology-Based Assessment of Sonazoid-Enhanced Ultrasonography for the Diagnosis of Liver Metastasis. *Ultrasound in Medicine and Biology* 2017; **43**(10): 2151-2158.

94. Liu J, Wang D, Li H, Li H, Zhou T, Zhao S *et al.* Clinical value of contrast-enhanced ultrasound in diagnosis of hyperechoic liver lesions. *Medical science monitor: international medical journal of experimental and clinical research* 2015; **21**: 2845.

95. Quaia E, De Paoli L, Angileri R, Cabibbo B, Cova MA. Indeterminate solid hepatic lesions identified on non-diagnostic contrast-enhanced computed tomography: assessment of the additional diagnostic value of contrast-enhanced ultrasound in the non-cirrhotic liver. *European journal of radiology* 2014; **83**(3): 456-462.

96. Sandrose S, Karstrup S, Gerke O, Rafaelsen S. Contrast Enhanced Ultrasound in CT-undetermined Focal Liver Lesions. *Ultrasound international open* 2016; **2**(4): E129.

97. Schellhaas B, Görtz RS, Pfeifer L, Kielisch C, Neurath MF, Strobel D. Diagnostic accuracy of contrast-enhanced ultrasound for the differential diagnosis of hepatocellular carcinoma: ESCULAP versus CEUS-LI-RADS. *European journal of gastroenterology & hepatology* 2017; **29**(9): 1036-1044.

98. Tada T, Kumada T, Toyoda H, Ito T, Sone Y, Kaneoka Y *et al.* Utility of contrast-enhanced ultrasound with perflubutane for diagnosing the macroscopic type of small nodular hepatocellular carcinomas. *European radiology* 2014; **24**(9): 2157-2166.

99. Thakur S, Jhobta A, Dhiman D, Sood R, Chauhan A, Thakur CS. Role of contrast enhanced ultrasound in characterization of focal liver lesions. *The Egyptian Journal of Radiology and Nuclear Medicine* 2014; **45**(1): 7-17.

100. Wang W-P, Dong Y, Cao J, Mao F, Xu Y, Si Q *et al.* Detection and characterization of small superficially located focal liver lesions by contrast-enhanced ultrasound with high frequency transducers. *Medical ultrasonography* 2017; **19**(4): 349-356.

101. Wu Jp, Shu R, Zhao Yz, Ma Gl, Xue W, He Qj *et al.* Comparison of contrast‐enhanced ultrasonography with virtual touch tissue quantification in the evaluation of focal liver lesions. *Journal of Clinical Ultrasound* 2016; **44**(6): 347-353.

102. Yin S, Cui Q, Yan K, Yang W, Wu W, Bao L *et al.* Effect of contrast-enhanced ultrasound on differential diagnosis of intrahepatic cholangiocarcinoma and arterial phase enhanced hepatic inflammatory lesions. *Chinese Journal of Cancer Research* 2017; **29**(3): 272.

103. Zhang P, Zhou P, Tian S-M, Qian Y, Li J-L, Li R-Z. Diagnostic Performance of Contrast‐Enhanced Sonography and Acoustic Radiation Force Impulse Imaging in Solid Liver Lesions. *Journal of Ultrasound in Medicine* 2014; **33**(2): 205-214.

104. Takahashi M, Maruyama H, Shimada T, Kamezaki H, Sekimoto T, Kanai F *et al.* Characterization of hepatic lesions (≤ 30 mm) with liver-specific contrast agents: a comparison between ultrasound and magnetic resonance imaging. *European journal of radiology* 2013; **82**(1): 75-84.

105. Taimr P, Jongerius VL, Pek CJ, Krak NC, Hansen BE, Janssen HL *et al.* Liver contrast-enhanced ultrasound improves detection of liver metastases in patients with pancreatic or periampullary cancer. *Ultrasound in Medicine and Biology* 2015; **41**(12): 3063-3069.

106. Cantisani V, Consorti F, Guerrisi A, Guerrisi I, Ricci P, Di Segni M *et al.* Prospective comparative evaluation of quantitative-elastosonography (Q-elastography) and contrast-enhanced ultrasound for the evaluation of thyroid nodules: preliminary experience. *European journal of radiology* 2013; **82**(11): 1892-1898.

107. Diao X, Zhan J, Chen L, Chen Y, Liu Y. Quantification of solid hypo-echoic thyroid nodule enhancement with contrast-enhanced ultrasound. *Translational Cancer Research* 2017; **6**(6): 1078-1087.

108. Giusti M, Orlandi D, Melle G, Massa B, Silvestri E, Minuto F *et al.* Is there a real diagnostic impact of elastosonography and contrast-enhanced ultrasonography in the management of thyroid nodules? *Journal of Zhejiang University SCIENCE B* 2013; **14**(3): 195-206.

109. Jiang J, Shang X, Wang H, Xu Y-B, Gao Y, Zhou Q. Diagnostic value of contrast-enhanced ultrasound in thyroid nodules with calcification. *The Kaohsiung journal of medical sciences* 2015; **31**(3): 138-144.

110. Wu Q, Wang Y, Li Y, Hu B, He Z-Y. Diagnostic value of contrast-enhanced ultrasound in solid thyroid nodules with and without enhancement. *Endocrine* 2016; **53**(2): 480-488.

111. Zhan J, Diao X-H, Chen L, Jin J-M, Chen Y. Role of Contrast-Enhanced Ultrasound in Diagnosis of Thyroid Nodules in Acoustic Radiation Force Impulse “Gray Zone”. *Ultrasound in Medicine and Biology* 2017; **43**(6): 1179-1186.

112. Zhang Y, Zhou P, Tian S-M, Zhao Y-F, Li J-L, Li L. Usefulness of combined use of contrast-enhanced ultrasound and TI-RADS classification for the differentiation of benign from malignant lesions of thyroid nodules. *European radiology* 2017; **27**(4): 1527-1536.

113. Zhang Y-Z, Xu T, Gong H-Y, Li C-Y, Ye X-H, Lin H-J *et al.* Application of high-resolution ultrasound, real-time elastography, and contrast-enhanced ultrasound in differentiating solid thyroid nodules. *Medicine* 2016; **95**(45).

114. Zhou X, Zhou P, Hu Z, Tian SM, Zhao Y, Liu W *et al.* Diagnostic Efficiency of Quantitative Contrast‐Enhanced Ultrasound Indicators for Discriminating Benign From Malignant Solid Thyroid Nodules. *Journal of Ultrasound in Medicine* 2018; **37**(2): 425-437.
