# Supplementary material for: Comparative Diagnostic Accuracy of Contrast-Enhanced Ultrasound and Shear Wave Elastography in Differentiating Benign and Malignant Lesions: A Network Meta-Analysis
Source: Front Oncol. 2019 Mar 5;9:102. doi: 10.3389/fonc.2019.00102 (PMC6412152; doi:10.3389/fonc.2019.00102)
Supplement: Supplementary File III — Additional Pairwise Meta-analysis Figures. [file Table_3.DOCX]

**Supplementary File III: Additional Pairwise Meta-analysis Figures**

1. **Breast**

**Meta-analysis of CEUS in breast**

**Meta-analysis of SWE in breast**

1. **Liver**

**Meta-analysis of CEUS in liver**

**Meta-analysis of SWE in liver**

1. **Thyroid**

**Meta-analysis of CEUS in thyroid**


**Meta-analysis of SWE in thyroid**

1. **Kidney**

**Meta-analysis of CEUS in kidney**


1. **Prostate**

**Meta-analysis of SWE in prostate**
